# Diagnostic and antimicrobial stewardship workforce challenges: A crisis in combating antimicrobial resistance

**DOI:** 10.1017/ash.2023.147

**Published:** 2023-03-29

**Authors:** Tristan T. Timbrook, Andrea M. Prinzi, Emily S. Spivak

**Affiliations:** 1 bioMérieux, Marcy l’Etoile, France; 2 University of Utah College of Pharmacy, Salt Lake City, Utah, United States; 3 Division of Infectious Diseases, Department of Internal Medicine, University of Utah School of Medicine, Salt Lake City, Utah, United States

*To the Editor—*Lost progress in the fight against antimicrobial resistance (AMR) is a frightening reality, and challenges in maintaining the healthcare infrastructure used to address the public health threat of AMR are increasingly prevalent. Shortages and attrition among clinical microbiologists, antimicrobial stewardship pharmacists, and physicians are common, particularly with the recent healthcare system strains of the coronavirus disease 2019 (COVID-19) pandemic.^1–3^ AMR was linked to 4.95 million global deaths in 2019, and the COVID-19 pandemic the following year made things dramatically worse.^4^ For instance, the Centers for Disease Control and Prevention (CDC) reported a 15% increase in antimicrobial-resistant infections in hospitals in 2020. At the same time, 80% of hospitalized patients with COVID-19 received an antibiotic between March and October 2020. Additionally, the CDC noted significant increases in infections with organisms like carbapenem-resistant *Acinetobacter* spp, which increased by 78% in 2020. AMR has the potential to undermine modern medicine and requires substantial immediate and sustained resources and action.^5^ The CDC recommends antimicrobial stewardship programs (ASPs) to combat AMR by ensuring the appropriate use of antimicrobials, and ASPs are most effective when co-led by infectious disease-trained pharmacists and physicians.^6^ Additionally, microbiologists are critical stakeholders in the success of this team in ensuring the ability to accurately and rapidly diagnose infections in addition to tracking AMR.^4,6^

Together, this team performs essential collaborations such as reporting antimicrobial resistance to the CDC, which is also critical to hospitals participating in the Promoting Interoperability Program by CMS Medicare as a condition of hospital participation.^7^ This frontline healthcare team is the backbone of the response against AMR yet faces substantial sustainability issues.

The COVID-19 pandemic highlighted how vital diagnostic testing and microbiologists are to healthcare systems and public health.^1^ Recent survey data have demonstrated increased vacancy rates for multiple medical laboratory areas and projected medical laboratory scientist retirement rates >20% for some departments.^8^ Filling vacancies was a top concern among many respondents (14.2%), exacerbated by COVID-19–related staffing challenges, inadequate salaries, the need for a heightened profile of the profession, and concerns about a lack of adequately trained staff.^1,8^ Importantly, staffing shortages in the medical laboratory are not benign and are associated with an increased risk of medical error.^1^ Furthermore, a reduction in adequately trained staff results in limitations in the extent of testing offered, leading to discontinuation of local testing and expanded off-site testing, which contributes to delays in appropriate treatment for patients.^1^

Lack of visibility and perceived lack of upward career mobility have been cited as drivers of laboratory staff retention, which could be addressed by further engaging the laboratory in stewardship efforts.^1^ For example, developing a diagnostic stewardship test utilization committee may help prevent burnout by reducing unnecessary testing while highlighting the important role of the laboratory in stewardship initiatives.^9^ In a recent survey of laboratorians in Canada demonstrated, 55% of respondents felt that involvement in stewardship initiatives was a valuable use of their time and they have an important role to play (70.6%). However, nearly 60% of respondents expressed that lack of time is a barrier driven by limited staffing and competing priorities.^9^ Although only one-third of hospitals in the United States report efforts to optimize diagnostic testing, this is 1 of the 6 leading stewardship practices and an area ripe for multidisciplinary improvement.^10^ In collaboration with stewardship personnel, microbiologists are responsible for guiding actions like diagnostic test selection, providing input on appropriate reporting, and following up on critical results. Optimizing existing test use and rapid diagnostic testing with adjunctive reporting algorithms can be crucial in building more sustainable and resilient healthcare systems for addressing AMR.

Like clinical laboratory scientists, chronic and pandemic-related acute issues threaten the sustainability of ASPs among pharmacists and physicians. A recent survey of ASPs reported an increase of 5 new duties related to COVID-19, whereas 18% of stewards noted less stewardship full-time equivalent (FTE) support during this period.^11^ Moreover, most stewards have noted less ability to perform traditional ASP activities, and 71% noted a positive screening for burnout.^11^ Beyond pandemic-related issues, attrition of clinical pharmacists has been noted related to a lack of ability to buy down time for nonclinical work (eg, committees, residency program director, etc), lack of capacity to produce revenue for activities, and limited career advancement opportunities.^3^ Similarly, supply challenges of infectious diseases physicians were highlighted in the 2020 fellowship match, where >1 in 5 US ID fellowships went unmatched.^2^ Reasons for this include being among the lowest-paid specialty due to revenue activities favoring procedure-based specialties, compounded by the significant financial burden of medical education, leading many to choose other specialties. These threats to recruiting and maintaining personnel with physicians and pharmacists are problematic because these leaders are critical to the success of ASPs.^6^ Survey data have suggested that for each 0.5 increase in pharmacist and physician FTE, an ∼1.5× increase in the effectiveness of ASPs occurs.^12^ In contrast, lower-resourced facilities (eg, rural and smaller hospitals) have been associated with less prospective audit and feedback, optimized diagnostic testing, and measurement of antibiotic use, which may contribute to rural health inequities in AMR.^10^ Robust and systematic investment in human resources is integral to the growth and sustainability of ASPs.

In conclusion, AMR is a critical public health issue, and the healthcare infrastructure and related policy should prioritize ensuring the support and sustainability of the ASP team as the frontline responders to AMR. As a clinical microbiologist, antimicrobial stewardship pharmacist, and infectious diseases physician, we see this as an urgent issue that must be addressed immediately to safeguard current and future progress in combating AMR.
